# Temporal Evolution of Parotid Volume and Parotid Apparent Diffusion Coefficient in Nasopharyngeal Carcinoma Patients Treated by Intensity-Modulated Radiotherapy Investigated by Magnetic Resonance Imaging: A Pilot Study

**DOI:** 10.1371/journal.pone.0137073

**Published:** 2015-08-31

**Authors:** Chun-Jung Juan, Cheng-Chieh Cheng, Su-Chin Chiu, Yee-Min Jen, Yi-Jui Liu, Hui-Chu Chiu, Hung-Wen Kao, Chih-Wei Wang, Hsiao-Wen Chung, Guo-Shu Huang, Hsian-He Hsu

**Affiliations:** 1 Department of Radiology, National Defense Medical Center, Taipei, Taiwan, Republic of China; 2 Department of Radiology, Tri-Service General Hospital, Taipei, Taiwan, Republic of China; 3 Graduate Institute of Biomedical Electronics and Bioinformatics, National Taiwan University, Taipei, Taiwan, Republic of China; 4 Department of Radiation Oncology, National Defense Medical Center, Taipei, Taiwan, Republic of China; 5 Department of Radiation Oncology, Tri-Service General Hospital, Taipei, Taiwan, Republic of China; 6 Department of Automatic Control Engineering, Feng-Chia University, Taichung, Taiwan, Republic of China; 7 Ph.D. program of Technology Management, Chung Hua University, Hsinchu, Taiwan, Republic of China; National Institutes of Health, UNITED STATES

## Abstract

**Purpose:**

To concurrently quantify the radiation-induced changes and temporal evolutions of parotid volume and parotid apparent diffusion coefficient (ADC) in nasopharyngeal carcinoma (NPC) patients treated by intensity-modulated radiotherapy by using magnetic resonance imaging (MRI).

**Materials and Methods:**

A total of 11 NPC patients (9 men and 2 women; 48.7 ± 11.7 years, 22 parotid glands) were enrolled. Radiation dose, parotid sparing volume, severity of xerostomia, and radiation-to-MR interval (RMI) was recorded. MRI studies were acquired four times, including one before and three after radiotherapy. The parotid volume and the parotid ADC were measured. Statistical analysis was performed using SPSS and MedCalc. Bonferroni correction was applied for multiple comparisons. A *P* value less than 0.05 was considered as statistically significant.

**Results:**

The parotid volume was 26.2 ± 8.0 cm^3^ before radiotherapy. The parotid ADC was 0.8 ± 0.15 × 10^−3^ mm^2^/sec before radiotherapy. The parotid glands received a radiation dose of 28.7 ± 4.1 Gy and a PSV of 44.1 ± 12.6%. The parotid volume was significantly smaller at MR stage 1 and stage 2 as compared to pre-RT stage (*P* < .005). The volume reduction ratio was 31.2 ± 13.0%, 26.1 ± 13.5%, and 17.1 ± 16.6% at stage 1, 2, and 3, respectively. The parotid ADC was significantly higher at all post-RT stages as compared to pre-RT stage reciprocally (*P* < .005 at stage 1 and 2, *P* < .05 at stage 3). The ADC increase ratio was 35.7 ± 17.4%, 27.0 ± 12.8%, and 20.2 ± 16.6% at stage 1, 2, and 3, respectively. The parotid ADC was negatively correlated to the parotid volume (*R* = -0.509; *P* < .001). The parotid ADC was positively associated with the radiation dose significantly (R^2^ = 0.212; *P* = .0001) and was negatively associated with RMI significantly (R^2^ = 0.203; *P* = .00096) significantly. Multiple regression analysis further showed that the post-RT parotid ADC was related to the radiation dose and RMI significantly (R^2^ = 0.3580; *P* < .0001). At MR stage 3, the parotid volume was negatively associated with the dry mouth grade significantly (R^2^ = 0.473; *P* < .0001), while the parotid ADC was positively associated with the dry mouth grade significantly (R^2^ = 0.288; *P* = .015).

**Conclusion:**

Our pilot study successfully demonstrates the concurrent changes and temporal evolution of parotid volume and parotid ADC quantitatively in NPC patients treated by IMRT. Our results suggest that the reduction of parotid volume and increase of parotid ADC are dominated by the effect of acinar loss rather than edema at early to intermediate phases and the following recovery of parotid volume and ADC toward the baseline values might reflect the acinar regeneration of parotid glands.

## Introduction

As a main treatment of choice for nasopharyngeal carcinoma (NPC), high-dose radiotherapy can cause structural damage and functional impairment of brain, brain stem, cranial nerve, basal skull, vessels, spinal cord, thyroid gland, and salivary glands [1–8]. Salivary glands are more susceptible to radiation than other organs due to their higher radiosensitivity, leading to xerostomia and reducing quality of life in patients after radiotherapy [9]. Mechanisms of radiation-induced damage of salivary glands have been investigated mostly in animal irradiation models, suggesting selective damage of plasma membrane of the secretory cells immediately after radiation exposure, followed by damage of DNA, death of acinar progenitor cells and finally lysis of acinar cells [10–14].

Compared to conventional radiotherapy, parotid-sparing radiotherapy techniques not only reduce the radiation dose delivered to parotid glands but also prevent these glands from permanent damages [15]. Animal studies have demonstrated regeneration of acinar cells following parotid atrophy induced by ductal obstruction [16] as well as by radiation exposure [17]. At the era of parotid-sparing radiotherapy, there is increasing demand for clinicians to be familiar with the radiation induced morphological and physiological changes of parotid glands and the temporal evolutions of these changes to see whether these changes are reversible or not. On the other hand, it is also important to evaluate radiotherapy-induced salivary gland injury in human directly since the human salivary glands may differ from the rat salivary glands in anatomic and physiological characteristics.

With the merit of noninvasiveness, imaging studies including computed tomography (CT) and magnetic resonance imaging (MRI) have been recently employed to investigate the radiation-induced damage of human salivary glands. Prior CT and MRI investigations have shown morphological [18–24], physiological [24–33], and functional [34, 35] changes of salivary glands after radiotherapy. Although radiation-induced volume changes [18–24] and diffusional alternations [28–33] of parotid glands have been recently examined separately, mechanism of injury and recovery of parotid glands following parotid sparing radiotherapy still cannot be completely explained by either morphological change or physiological change alone. For example, reduced volume of parotid glands might be due to acinar loss [12, 13] or fibrosis [13], while increased volume might be due to acute inflammation [36] or lipomatosis [37, 38]. Besides, increased ADC might be due to acute inflammation or increased extravascular extracellular space due to cellular loss, while decreased ADC might be due to recovery from acute inflammation, increased cellularity [39] or abundant fatty component [40].

To the best of our knowledge, concurrent evaluation of changes and temporal evolutions of parotid volume and ADC after radiotherapy has not been documented to date. We hypothesized that MRI is capable of concurrently and noninvasively delineating the changes and temporal evolutions of parotid volume and ADC in patients after parotid sparing radiotherapy and probing the mechanisms of parotid injury and recovery after parotid sparing radiotherapy. In this study, we aimed to quantify the radiation-induced changes and temporal evolutions of parotid volume and ADC concurrently in NPC patients treated by intensity-modulated radiotherapy (IMRT) and to discuss the underlying mechanism of radiation injury and recovery process of the parotid glands.

## Methods and Materials

This study was approved by the institutional review board of Tri-Service General Hospital. Written informed consents were waived for this retrospective study, in which patient information was anonymized and de-identified after imaging processing and prior to analysis.

### Patients

Since July 2008 to April 2009, 11 patients (9 men and 2 women; 48.7 ± 11.7 years; 22 parotid glands) who were newly diagnosed as NPC, treated by IMRT, and received pre-radiotherapy MRI studies and at least two post-radiotherapy (post-RT) follow-up MRI studies were recruited in this retrospective study. All the enrolled patients were free from any parotid lesion or prior operation in head and neck.

Severity of xerostomia was evaluated by a 20-year experienced radiation oncologist (J.Y.M.) using a Radiation Therapy Oncology Group (RTOG) five-point system first described by Cox et al. [41]. The grades of xerostomia ranged from 0 to 4 (grade 0 represents no symptom of dry mouth; grade 1 represents slight dryness of the mouth, good response on stimulation; grade 2 represents moderate dryness, poor response on stimulation; grade 3 represents complete dryness, no response on stimulation; grade 4 represents necrosis of the salivary glands). Parotid sparing volume (PSV), defined as the fractional parotid gland volume receiving a radiation dose than less 25 Gy, was calculated based on the dose volume histogram.

### Image acquisition

All MR examinations were performed at a 1.5T scanner (GE Healthcare, Signa HDx, US) with an 8-channel head-and-neck coil. MR images including axial T2-weighted images (T2WI) and diffusion-weighted images (DWI) were acquired for evaluation of morphology and diffusion of parotid glands, respectively. On T2WI, a fast spin-echo (FSE) sequence (TR/TE/ NEX = 3150 ms/80 ms/2) was used using an echo train length of 22, a field of view of 240 × 240 mm, a matrix size of 512 × 512, an in-plane resolution of 0.47 × 0.47 mm^2^ and a slice thickness was 5 mm with an inter-slice gap of 1 mm. On DWI, a single-shot echo-planar imaging sequence (TE/TR/NEX = 73.3ms/7000ms/4) was acquired with diffusion gradients (0 and 1000 sec/mm^2^) applied along three orthogonal axes, a field of view of 240 × 240 mm, a matrix size of 256 × 256, an in-plane resolution of 0.94 × 0.94 mm^2^ and a slice thickness was 5 mm with an inter-slice gap of 1 mm. A total of 18 slices with a slice thickness of 5 mm were acquired to cover from nasopharynx to the parotid glands.

For the sake of longitudinal evaluation of parotid glands, MRI studies were categorized into 4 stages according to the timing of MR examinations. Stage 0, 1, 2 and 3 represented the timing when MRI studies were obtained before radiotherapy, within 100 days after radiotherapy, between 101 days to 1 year after radiotherapy and more than 1 year after radiotherapy, respectively. The overall radiation-to-MR interval (RMI) was 51.2 ± 15.9 days, 240.3 ± 54.6 days, and 489.3 ± 99.2 days for stage 1, 2 and 3, respectively. Except one patient who refused chemotherapy, all others were treated by concurrent chemoradiotherapy (CCRT). Two patients expired before the third post-RT MRI studies. Hence, a total of 42 MRI examinations were obtained and analyzed.

### Data analysis

**Fig 1:**
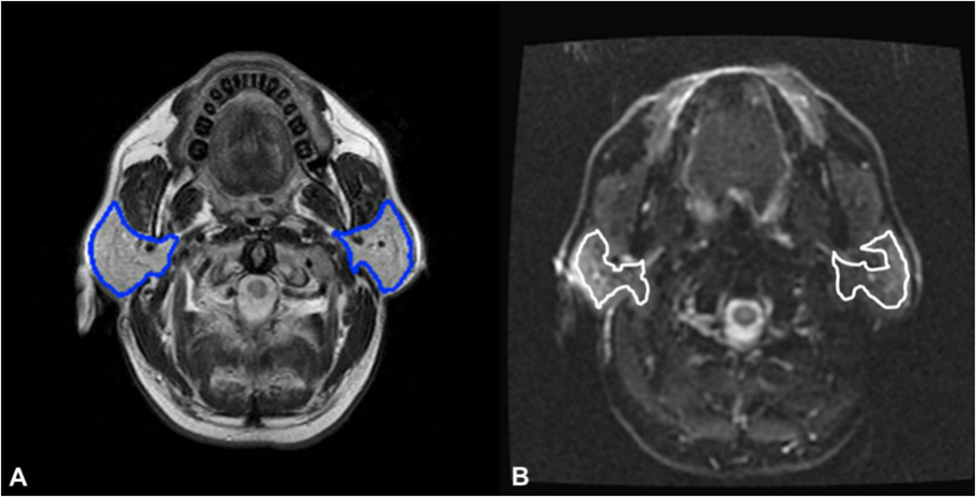
T2WI and DWI of a patient with demonstration of ROI drawing for parotid glands. Parotid glands are contoured (blue polygons) on T2WI for volume measurement (1A). For measurement of parotid ADC, ROIs are drawn about 2-mm medially to the margin of glands with excluding visible vessels (while polygons) on DWI to avoid partial volume effect (1B).

All MR images following the digital imaging and communications in medicine format were transferred to a personal computer. MR images were processed using software developed in-house (C.C.C.) on Matlab platform (MathWorks, Natick, Mass). For volume measurements, parotid glands were manually contoured (Fig 1A) slice-by-slice on T2WI by C.C.C. (2 years of experience in head and neck MR imaging investigation) in consensus by C.J.J. (more than 5 years of experience in head and neck MR imaging interpretation). The parotid volume was calculated according to the Eq 1:

$$V=\underset{i}{\sum }Ai\times \left(ST+1\right)$$ [1],

where *V* represented volume of the parotid gland, *A*_*i*_ represented area of parotid gland in *i*th slice and *ST* represented slice thickness.

For measurement of apparent diffusion coefficient (ADC), ADC maps were generated first by pixel-by-pixel calculation based on the Eq 2:

$$\text{ADC}=\frac{In\;\left(SI_{0}/SI_{1000}\right)}{b_{1000}-b_{0}}$$ [2]

,where SI_0_ and SI_1000_ represent the signal intensities of the DWI obtained using diffusion gradients (b values) of 0 sec/mm^2^ (b_0_) and 1000 sec/mm^2^ (b_1000_), respectively. To avoid the partial volume effect of surrounding tissues and vessels, polygonal regions-of-interest (ROIs) of parotid glands were manually drawn on DWI (b_1000_) with about a 2-mm distance inward the margin of the glands with excluding visible vessels (Fig 1B). Mean ADC was calculated by averaging the ADC values of all pixels within the ROIs of each parotid gland. In order to provide a normalized trend, ADC increase ratio as defined by relative increase of post-RT ADC to pre-RT values was calculated. Likewise, volume reduction ratio as defined by relative reduction of post-RT volume to pre-RT values was also computed.

### Statistical analysis

Statistical analyses were performed by using SPSS 16.0 (SPSS, Chicago, Ill) and MedCalc 13.0.4.0 (MedCalc, Mariakerke, Belgium). Normality of parotid volume and parotid ADC was examined by Kolmogorov-Smirnov tests. Wilcoxon Signed Ranks Test was used to examine the difference between radiation dose delivered to the right and left parotid glands and the difference of PSV on either side. Mann-Whitney test was used to examine the difference of radiation dose delivered to the parotid glands and the difference of PSV at different T stages. Analysis of variance (ANOVA) and two-tail paired T-test was utilized for comparing the volume and ADC of parotid glands among different stages of MRI after Bonferroni correction for multiple comparisons. Linear regression analysis test was applied on the relationship between radiation dose and PSV, the relationship between parotid volume and parotid ADC, the relationship between parotid volume (parotid ADC) and radiation dose (PSV and RMI). A *P* value less than 0.05 was considered as statistically significant.

## Results

The radiation dose delivered to the tumor was 70.5 ± 1.3 Gy (mean ± standard deviation). The overall radiation dose transmitted to parotid glands (28.7 ± 4.1 Gy) was significantly smaller than to the tumor (*P* < .005), ranging from 21.8 Gy to 40.5 Gy. The overall PSV of 44.1 ± 12.6% was achieved, ranging from 18% to 69.1%. The radiation dose delivered to parotid glands was of no significant difference (*P* > .5) among patients at T2 (28.0 ± 2.6 Gy), T3 (28.1 ± 3.2 Gy), and T4 (30.0 ± 5.7 Gy) stages. The PSV was of no significant difference (*P* > .08) among patients at T2 (46.0 ± 7.1%), T3 (51.3 ±10.4%), and T4 (37.0 ± 15.6%) stages, either.

The radiation dose and the PSV were significantly different between right parotid glands and left ones due to pre-RT planning intentionally. The right parotid glands obtained a radiation dose (30.6± 3.9 Gy) significantly higher than that (26.9 ± 3.5 Gy) of the left parotid glands (*P* < .005). On the contrary, the PSV on the right (39.9± 11.5%) was significantly lower than that on the left (48.3± 12.8%) (*P* < .005). Linear regression analysis disclosed that the PSV had a significant negative correlation with the radiation dose delivered to parotid glands (y = -1.738x + 94) with a correlation coefficient (*R*) of -0.56 (*P* < .01). Because of significant difference of radiation dose delivered to the right and left parotid glands (*P* < .005), each parotid gland was treated as an individual gland in the following statistical analysis with respect to parotid volume and parotid ADC in this study.

**Fig 2:**
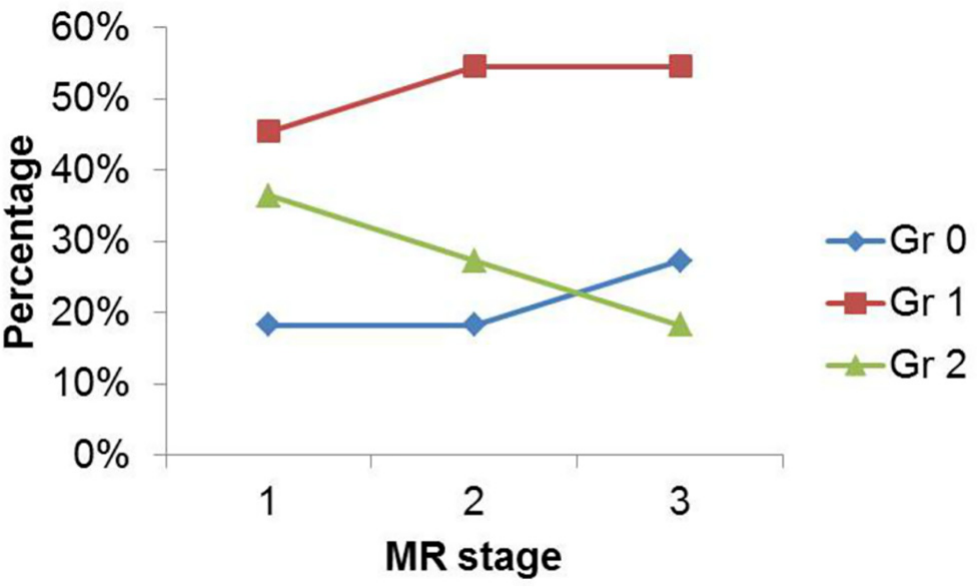
Percentage of dry mouth grade versus MR stage, showing continuous decrease of grade 2 xerostomia percentage as MR stage increased.

The percentage of dry mouth grade versus MR stage was demonstrated in Fig 2. The percentage of grade 2 xerostomia continuously decreased as MR stage increased. In MR stage 1, 18.2% (2 of 11) of patients encountered grade 0 xerostomia, 45.4% (5 of 11) experienced grade 1 xerostomia, 36.4% (4 of 11) had 2 xerostomia. In MR stage 3, 27.3% (3 of 11) of patients encountered grade 0 xerostomia, 54.5% (6 of 11) experienced grade 1 xerostomia, and 18.2% (2 of 11) patients had grade 2 xerostomia.

### Morphology and volume of parotid glands versus MR stages

**Fig 3:**
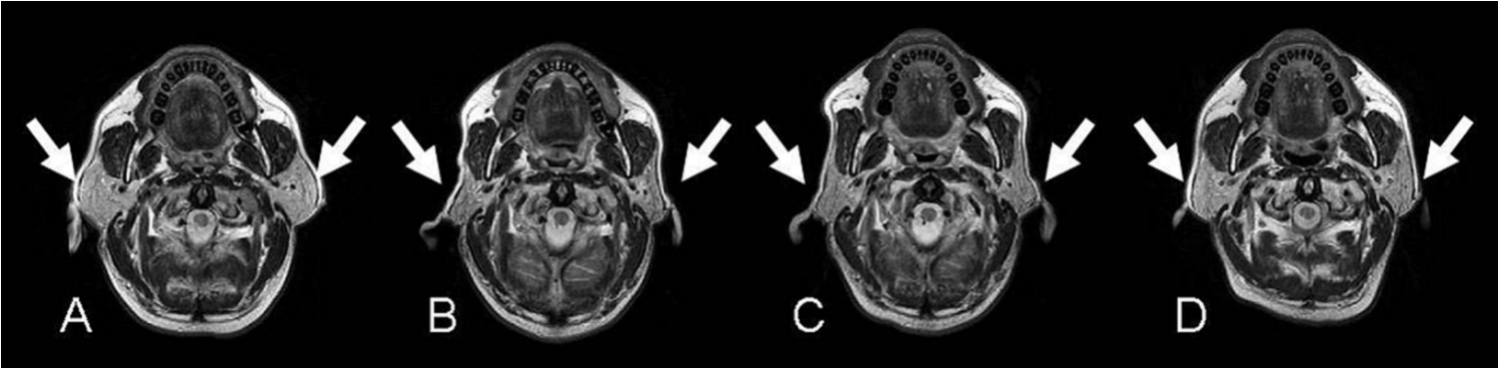
A 54-year-old patient with nasopharyngeal carcinoma with a radiation dose to the tumor, right parotid gland and left parotid gland of 72 Gy, 26.49 Gy and 24.75 Gy, respectively. Serial axial T2-weighted images of were obtained at MR stage 0 (3A; before radiotherapy), 1 (3B; 53 days), 2 (3C; 270 days) and 3 (3D; 435 days), respectively. The parotid glands (arrows) showed perceivable shrinkage at stage 1 followed by gradual restoration at stage 2 and stage 3.

The parotid volume was 26.2 ± 8.0 cm^3^ before radiotherapy. Fig 3 illustrated the morphological evolution of the parotid glands in one patient. The outer margins of parotid glands were convex before radiotherapy (Fig 3A), became concave at day 53 after radiotherapy (Fig 3B), appeared flat at day 270 after radiotherapy (Fig 3C), and returned to convex at day 435 after radiotherapy (Fig 3D).

**Fig 4:**
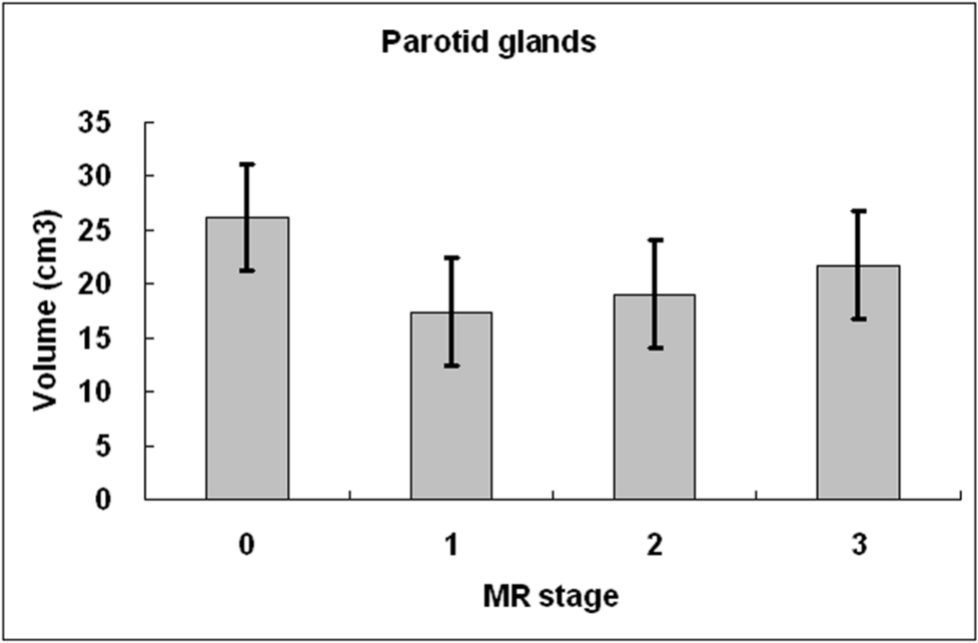
Parotid volume at different MR stages in nasopharyngeal carcinoma patients treated by intensity-modulated radiotherapy. Compared to MR stage 0 (before radiotherapy), parotid volume reduced significantly at stage 1 (≦100 days) and stage 2 (101 days to 1 year). The parotid volume is smallest at stage 1 with gradual increase at stage 2 and stage 3 (>1 year).

Fig 4 demonstrated absolute parotid volume with respect to MR stages. The parotid volume was significantly smaller at stage 1 (*P* < .005) and stage 2 (*P* < .005) as compared to the pre-RT stage, respectively. At stage 3, the parotid volume was still smaller than that at pre-RT stage, however, the difference was not significant (*P* = .56). The post-RT parotid volume was smallest at stage 1 followed by stage 2 and stage 3 in an increasing order (*P* = .088 to 1). The volume reduction ratio was 31.2 ± 13.0%, 26.1 ± 13.5%, and 17.1 ± 16.6% at stage 1, 2, and 3, respectively.

### ADC of parotid glands versus MR stages

**Fig 5:**
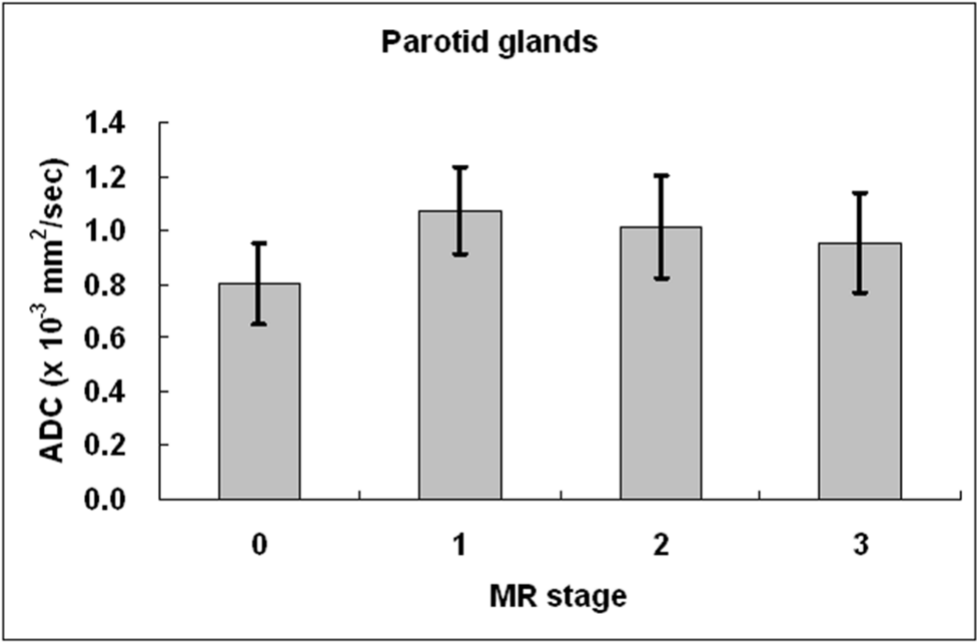
Parotid ADC at different MR stages in nasopharyngeal carcinoma patients treated by intensity-modulated radiotherapy. Compared to stage 0 (before radiotherapy), parotid ADC increased significantly at all MR stages. After initial elevation at stage 1 (≦100 days), parotid ADC gradually decreased at stage 2 (101 days to 1 year) and stage 3 (>1 year).

The parotid ADC before radiotherapy was 0.8 ± 0.15 × 10^−3^ mm^2^/sec. Fig 5 showed the quantitative changes of parotid ADC with respect to MR stages. The parotid ADC was significantly higher at stage 1 (*P* < .005) and stage 2 (*P* < .005) than at the pre-RT stage, respectively. At stage 3, although the parotid ADC was still higher than that at pre-RT stage, the difference was not significant (*P* = .153). The post-RT parotid ADC was highest at stage 1 followed by stage 2 and stage 3 in a decreasing order, with significant difference between stage 1 and stage 3 (*P* < .05). The ADC increase ratio was 35.7 ± 17.4%, 27.0 ± 12.8%, and 20.2 ± 16.6% at stage 1, 2, and 3, respectively.

### Relationship of parotid ADC versus parotid volume

**Fig 6:**
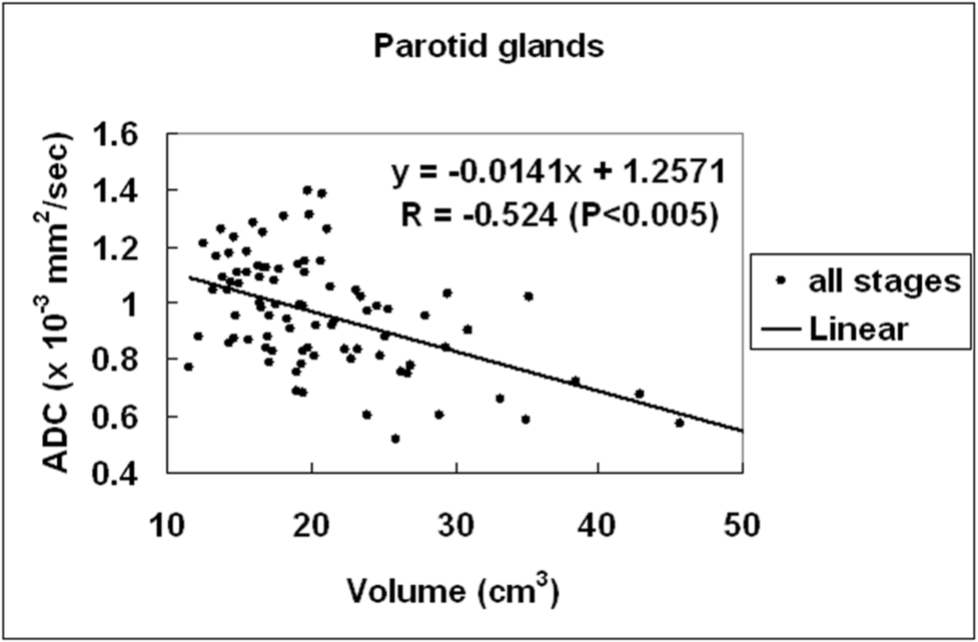
Scatter plots of parotid ADC versus parotid volume. Linear regression of parotid ADC versus parotid volume at all MR stages shows that the parotid ADC is negatively correlated to the parotid volume with significance (*P* < .005).

Linear regression analysis disclosed that the parotid ADC was negatively correlated to the parotid volume (y = -0.0122 + 1.245x × 10^−3^ mm/sec^2^) with significance (R^2^ = 0.1738; *P* = .0006) (Fig 6).

### Parotid volume and parotid ADC among different dry mouth grades

**Fig 7:**
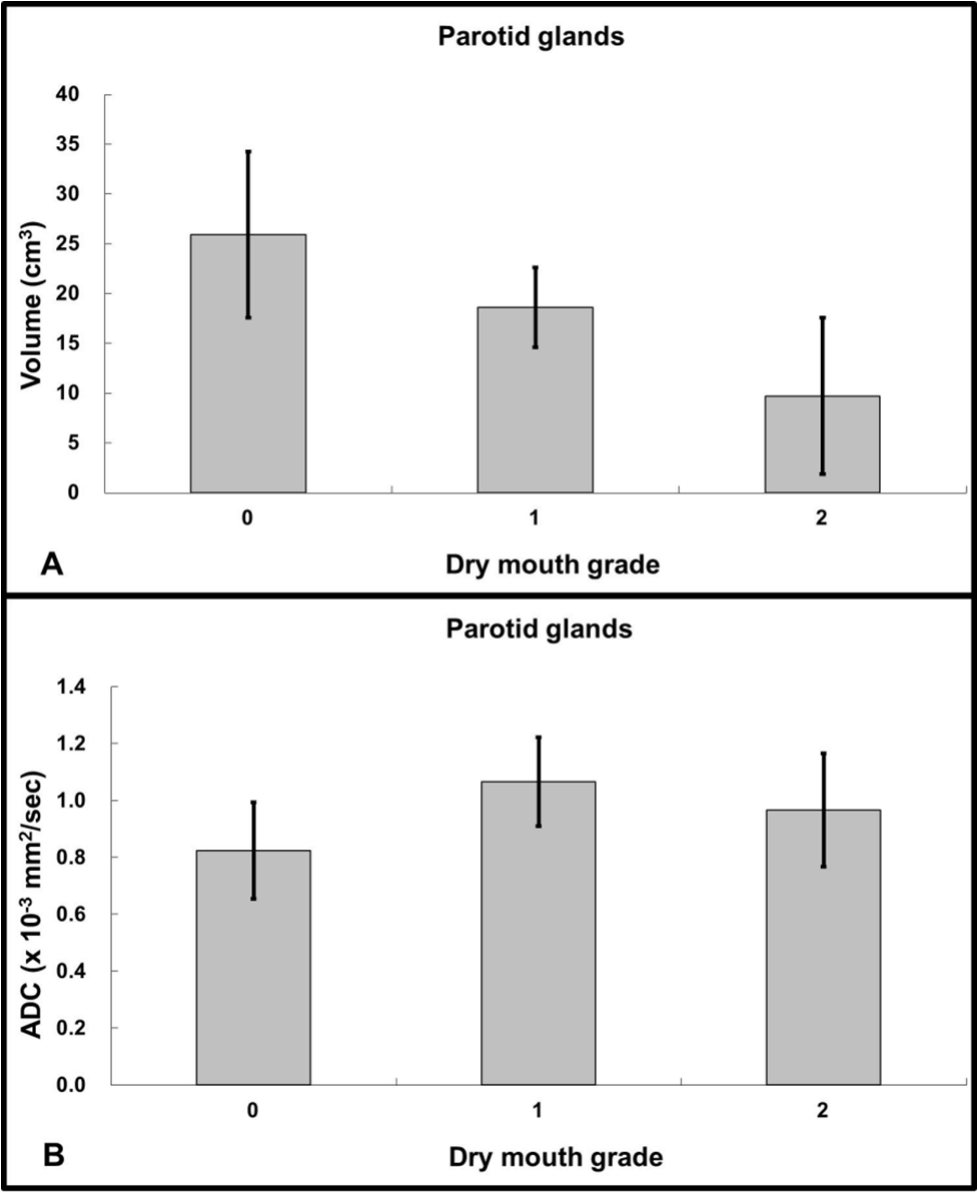
Parotid volume versus xerostomia (7A) and parotid ADC versus xerostomia (7B) in nasopharyngeal carcinoma patients treated by intensity-modulated radiotherapy. The parotid volume is significant lower in grades 1 and 2 as compared to grade 0 (*P* < .005). On the contrary, the parotid ADC was significant higher in grades 1 and 2 as compared to grade 0 (*P* < .005). Note: Grade 0 represents no symptom of dry mouth; grade 1 represents slight dryness of the mouth with good response on stimulation; grade 2 represents moderate dryness with poor response on stimulation.

Parotid volume and parotid ADC in each dry mouth grade were graphically shown on Fig 7. The parotid volume was significantly smaller at grade 1 (*P* < .005) and grade 2 (*P* < .005) as compared to that at grade 0, respectively. The parotid volume at grade 1 did not differ from that at grade 2 (*P* = .187). The parotid ADC was significantly higher at grade 1 (*P* < .005) and grade 2 (*P* < .005) as compared to that at grade 0, respectively. The parotid ADC at grade 1 did not differ from that at grade 2 (*P* = 1). At MR stage 3, the parotid volume was negatively associated with the dry mouth grade significantly (y = 29.201–8.326x ml; R^2^ = 0.473; *P* < .0001). On the contrary, the parotid ADC was positively associated with dry mouth grade significantly (y = 0.828 + 0.138x ×10^-3^mm/sec^2^; R^2^ = 0.288; *P* = .015).

### Parotid volume and ADC as a function of radiation dose, PSV, and RMI

Linear regression analysis showed that the parotid volume was not related to any of the radiation dose (R^2^ = 0.016; *P* = .31), PSV (R^2^ = 0.082; *P* = .11), and RMI (R^2^ = 0.0002; *P* = .92). The parotid ADC was positively associated with the radiation dose significantly (y = 0.409 + 0.021x ×10^-3^mm/sec^2^; R^2^ = 0.212; *P* = .0001) and was negatively associated with RMI significantly (y = 1.1522–0.00042x ×10^-3^mm/sec^2^; R^2^ = 0.203; *P* = .00096) significantly. The association between the parotid ADC and PSV was not significant (R^2^ = 0.045; *P* = .092). Multiple regression analysis further showed that the post-RT parotid ADC was related the radiation dose and RMI significantly (y = 0.541 + 0.01989 radiation dose– 0.0003847 RMI ×10^-3^mm/sec^2^; R^2^ = 0.3580; *P* < .0001).

## Discussion

Radiotherapy usually causes salivary gland injury and complicates with xerostomia. With a high dose of 50 to 60 Gy delivered to the parotid glands, 64% of the patients encounter grade 2 xerostomia [42]. With a mean radiation dose of 28.7 Gy and PSV of 44.1%, most patients (63.6%) of our group encountered grade 0 or grade 1 xerostomia, while only 36.4% were suffered from grade 2 xerostomia at early-to-intermediate stage after radiotherapy. Our results were in consistent with Nishimura’s study, in which 57.5% of patients have grade 0 or grade 1 xerostomia, while 42.5% have grade 2 or grade 3 xerostomia at 3 months after a mean dose of 27.2 Gy of radiotherapy [22].

Morphological change of human parotid glands after radiotherapy can be evaluated noninvasively by using either CT or MRI. Prior studies have depicted that high dose (70 Gy) of radiation leads to as much as 41% of parotid volume reduction [18], while lower dose of radiation causes less parotid volume reduction, i.e. 30.0% to 35.4% loss of parotid volume after 30 Gy [19, 20], 27.2% loss after 22.2 Gy [21], and 25.8% loss after 27.2 Gy [22] of irradiation. Our results, showing 31.2% of parotid volume reduction after 28.8 Gy of radiotherapy, are in consistent with prior researches with low dose radiation.

The radiation damage of salivary gland has been previously classified into four phases, including acute phase (0–10 days), early phase (10–60 days), intermediate phase (60–120 days) and late phase (120–240 days) [43]. For comparison, a chronic phase (>240 days) to Coppes’ classification was added in this study. Whether the parotid volume loss after parotid sparing radiotherapy is transient or permanent is of increasing interest and is getting more attention recently. Longitudinal changes of parotid volume after irradiation have not been documented until 2009, when Wang et al reported an averaged volume loss of 20.0% during, 26.9% at the end, and 27.2% at 2 months after radiotherapy [21]. Their results suggest that parotid volume reduction occurs as early as during the radiotherapy. In 2011, Tomitaka et al documented a 30.5% parotid volume reduction at 2 months, a maximal volume loss (35.4%) at 6 months, and gradual recuperation of parotid volume up to two years after 30 Gy of radiotherapy [20]. Tomitakas’ results imply that the parotid volume loss reaches the maximum at late stage. Our study shows significant volume reduction (31.2%) at 51 days after radiotherapy, followed by 26.1% of volume reduction at 240 days, and 17.1% of volume reduction at 489 days. Our results suggest that the parotid volume loss may reach the maximum at early to intermediate phase. Nevertheless, the gradual restoration of parotid volume at later phases after parotid sparing radiotherapy is found by both Tomitakas’ and our studies consistently.

The radiation-induced change of parotid ADC has been investigated since 2001, when Zhang et al reported a reduction of parotid ADC from 2.48 × 10^−3^ mm^2^/sec to 1.91 × 10^−3^ mm^2^/sec after 55.2 Gy of irradiation [28]. In 2005, however, Studer et al presented a discrepant result by showing increase of parotid ADC from 1.0 × 10^−3^ mm^2^/sec to 1.49 × 10^−3^ mm^2^/sec at the end of radiotherapy [33]. Later in 2008, Dirix et al documented an increase of parotid ADC (1.08 × 10^−3^ mm^2^/sec) after high dose (53.9 Gy) of radiotherapy as compared to that (0.84 × 10^−3^ mm^2^/sec) before radiotherapy but no change of parotid ADC (0.85 × 10^−3^ mm^2^/sec) after low dose (20.0 Gy) of radiotherapy [29]. In 2013, Zhang Y et al also disclosed significant increase of parotid ADC after radiotherapy [30]. The diffusion gradients (b values) are different in these studies. While Zhang L et al used low b values (10 to 150 sec/mm^2^), the others used high b values (400~ 500 to 1000 sec/mm^2^). Prior researches have shown that the parotid ADC is influenced by b values with a negative association [44]. The low b values used by Zhang L et al are believed to encode the fast water motion known as perfusion, while the high b values used by other researchers are widely accepted to encode the slow water motion known as diffusion. By using b values of 0 and 1000 sec/mm^2^, our study discloses an increase of parotid ADC from 0.80 × 10^−3^ mm^2^/sec to 1.07 × 10^−3^ mm^2^/sec after radiotherapy. Our results are in consistent with prior studies using high b values regarding the baseline ADC of parotid glands and the increase of parotid ADC after radiotherapy [29, 30, 33]. In addition to b values, the parotid ADC can also be influenced by lots of factors, including pulse sequences, accelerating factors, histological components, fat saturations, and bulk motions [40, 45, 46].

In the brain, increase of ADC has been observed in either vasogenic edema [47] or cellular lysis [48]. It is reasonable that the increase of parotid ADC after radiotherapy can be due to either edema or acinar lysis. Acute inflammation is often accompanied by swelling of parotid glands [49], while acinar loss is usually associated with shrinkage of parotid glands [12, 14]. Whether the increase of parotid ADC after radiotherapy is caused by radiation-induced acute inflammation or acinar loss remains unclear. In our study, the findings of concurrent increase of parotid ADC and reduction of parotid volume at early to intermediate phases suggest an increase of interstitial space subsequent to radiation-induced acinar loss rather than acute inflammation. On the other hand, reduction of parotid volume might be due to either acinar loss or fibrosis. In liver study, the fibrotic livers have shown to have lower ADC than the nonfibrotic livers [50]. Accordingly, the concurrent increase of ADC suggests that the reduction of parotid volume is mainly due to acinar loss rather than fibrosis in our study.

Longitudinal change of parotid ADC after radiotherapy has not been documented until 2005, when Studer et al presented serial ADC measurements before, during, and at 6 weeks after radiotherapy in a conference proceeding [33]. Studers’ results show an increase of ADC at the end of radiotherapy but without additional change at early phase (6 weeks) after radiotherapy. Our study uncovers the serial ADC changes of parotid glands from early to chronic phases, showing significant increase (35.7%) of parotid ADC at early-to-intermediate phase, followed by gradual reduction of parotid ADC at late phase (27.0%) and chronic phase (20.2%).

The reciprocal changes of increased parotid ADC and reduced parotid volume are observed at all phases. Our results further disclose a significant negative correlation between the parotid ADC and the parotid volume. Such negative association between parotid ADC and parotid volume, again, supports that the change of parotid ADC at early to chronic phases after radiotherapy is dominated by the effect of acinar numbers rather than inflammation. Furthermore, we also found that the parotid ADC is positively associated to the radiation dose and negatively associated to RMI significantly. On the contrary, there was no association between the parotid volume and the radiation dose and between the parotid volume and the RMI.

Our results show that patients with grade 1 and grade 2 xerostomia have significantly smaller parotid volume and significantly higher ADC than those without xerostomia. At MR stage 3, the parotid volume was negatively associated with the dry mouth grade significantly, while the parotid ADC was positively associated with the dry mouth grade significantly. Our results provide a link among morphological (volume), physiological (ADC) and functional (xerostomia) alternations of parotid glands after radiotherapy.

Our study has some three potential limitations. First, the sample size of this pilot study is relatively small. However, our pilot study analyzes a total of 22 different parotid glands at 4 different MR examinations. It is believed to shed a light on the understanding of the potential mechanism of radiation injury to the parotid glands. To verify these findings, we have launched another study to recruit more patients. Second, the salivary flow is not evaluated in this study. By comparing to the dry mouth grade, our results also correlate the parotid volume and the parotid ADC the parotid function (represented dry mouth grade) indirectly. Finally, we did not evaluate the regional characteristics of parotid volume and ADC. Although regionally dependent radiosensitivity of parotid glands has been observed in few rat studies [51], it remains unclear whether there is similar regionally dependent radiosensitivity in human parotid glands. In addition, the signal intensities of the parotid glands in our images were rather homogeneous visually. We believe that the imaging features are less likely to be strongly regionally dependent.

In conclusion, our pilot study successfully demonstrates the concurrent changes and temporal evolutions of parotid volume and parotid ADC in NPC patients treated by IMRT. Our results suggest that the reduction of parotid volume and increase of parotid ADC are dominated by the effect of acinar loss rather than edema at early to intermediate phases and the following recovery of parotid volume and ADC toward the baseline values might reflect the acinar regeneration of parotid glands.

## Supporting Information

S1 Data Clinical data and MRI measurements in this study. (XLSX)
